# Development of a Low-Cost Attitude and Heading Reference System Using a Three-Axis Rotating Platform

**DOI:** 10.3390/s100402472

**Published:** 2010-03-24

**Authors:** Ying-Chih Lai, Shau-Shiun Jan, Fei-Bin Hsiao

**Affiliations:** Institute of Aeronautics and Astronautics, National Cheng Kung University, Tainan, 701, Taiwan; E-Mails: p4893126@ccmail.ncku.edu.tw (Y.-C.L.); fbhsiao@mail.ncku.edu.tw (F.-B.H.)

**Keywords:** attitude and heading reference system (AHRS), calibration, micro electro-mechanical system (MEMS), anisotropic-magnetoresistive (AMR)

## Abstract

A development procedure for a low-cost attitude and heading reference system (AHRS) with a self-developed three-axis rotating platform has been proposed. The AHRS consists of one 3-axis accelerometer, three single-axis gyroscopes, and one 3-axis digital compass. Both the accelerometer and gyroscope triads are based on micro electro-mechanical system (MEMS) technology, and the digital compass is based on anisotropic-magnetoresistive (AMR) technology. The calibrations for each sensor triad are readily accomplished by using the scalar calibration and the least squares methods. The platform is suitable for the calibration and validation of the low-cost AHRS and it is affordable for most laboratories. With the calibrated parameters and data fusion algorithm for the orientation estimation, the self-developed AHRS demonstrates the capabilities of compensating for the sensor errors and outputting the estimated orientation in real-time. The validation results show that the estimated orientations of the developed AHRS are within the acceptable region. This verifies the practicability of the proposed development procedure.

## 1. Introduction

The orientation of a vehicle in three-dimensional space is one of the most significant pieces of information required for the navigation, guidance and control of that vehicle. The attitude and heading reference system (AHRS) is a general device to determine the orientation of a vehicle or an object which it is attached to. Recently, investigations of attitude estimation with low-cost sensors based on micro electro-mechanical system (MEMS) have been conducted [1,2]. The features of MEMS sensors are their light weight and small size, hence their applications such as small unmanned aerial vehicles (UAVs) and in human body motion tracking, *etc.*, are widespread. However, these low-cost sensors suffer from large noise and errors, and this is the reason why the calibration and validation of the AHRS based on low-cost sensors are critical and necessary procedures to verify its accuracy and performance before its implementation.

There are many calibration methods for the inertial measurement unit (IMU), which mainly consists of the accelerometers and gyroscopes. The multi-position and rate tests are the common methods that involve mounting the unit on a precision three-axis table [3]. These tests are undertaken by rotating the unit to a series of accurately known angles and positioning it in different orientations with respect to the local gravity vector. Another similar method is applied on a mechanical platform to perform 18 precise and specific orientations, while the angular rate between orientations is maintained constant and known [4]. Since these methods require high precision equipment, some other methods have been developed to calibrate the MEMS inertial sensors and the magnetometers based on the anisotropic-magnetoresistive (AMR) technology. An algorithm called scalar calibration has been used to calibrate low-cost accelerometers and magnetometers in various random orientations in homogeneous gravity and magnetic fields [5]. By using this method, the nine parameters—three scale factors, three biases and three nonorthogonal angles—for each sensor triad can be determined. The least squares method is the algorithm commonly used in the scalar calibration to estimate the calibration parameters [6–9]. For the calibration of the low-cost gyroscopes, the Earth rotation rate is smaller than its resolution; therefore, there are two solutions to this problem. The first one adopts a turntable to generate desired angular rate [9–11], while the other one performs the orientation estimation from angular rate integration *via* mathematical reasoning [7,8].

In order to evaluate the accuracy of the calibrated parameters and to validate the developed AHRS, some devices and methods are required. For example, an optical kinematic measurement system had been applied to evaluate the performance of the AHRS developed in [7]. Another method is to apply the calibrated parameters to the field tests of the navigation system with the integration of the inertial navigation system (INS) and global positioning system (GPS) as presented in [9–11]. A three-axis platform with angular position feedback is another alternative to achieve the validation of the calibrated parameters [12].

Almost all of the calibration and validation methods mentioned previously require either a precise platform or complicated procedures. For the development of a low-cost AHRS, the accuracy is not a significant issue, but the reliability and practicability are of greater concern. From our experience, the acceptable attitude errors for the navigation of a small UAV are within 3° [13]. Therefore, a three-axis rotating platform with acceptable precision is adequate to calibrate and validate the low-cost AHRS. For this reason, the goal of this study is to perform a convenient, simple and straightforward method for the development, calibration and validation of a low-cost AHRS by using a three-axis rotating platform. The calibration of this AHRS contains two stages. The first one is the calibration of the sensor triads and the second is to calibrate the output angles from the AHRS. The calibrations of the sensor triads are done by collecting data to assess the performance of an existing calibration approach. The purpose of the validation procedure is to evaluate the performance of the AHRS. In order to achieve the goal of this study, the requirements of this platform are that it be capable of angular position and rate feedback for each axis and have the ability of simulating the dynamic motion of the object the AHRS is attached to. One important issue of the AHRS design is its dynamic response, which is based on the application scope. With this ability, the design and validation of the AHRS will become more convenient. The precision of this platform is not critical due to the implementation of the low-cost sensors in the AHRS and the application of the scalar calibration method to the sensor error calibration. With the calibrated parameters and the applied data fusion algorithm, the estimated orientation of the developed AHRS can be obtained. Then the performance of this AHRS is validated through the above mentioned platform.

## 2. Low-Cost AHRS Design

### 2.1. Hardware Overview

In general, the orientation of the AHRS is derived from the inertial sensors, *i.e.*, accelerometers and gyroscopes, and the magnetic sensors, magnetometers. In this study, the AHRS consists of one 3-axis ADXL 330 accelerometer, three single-axis ADXRS300 gyroscopes, and one 3-axis HMC2003 digital compass which consists of one single-axis and one dual-axis magnetometers. The full-scale range of the accelerometer and the gyroscopes are ±3 g and ±300°/s, respectively. Both these inertial sensors are based on MEMS technology and are produced by Analog Devices. The digital compass is based on AMR technology and produced by Honeywell. The full-scale range of the digital compass is ±2 gauss. Although the digital compass is termed a 3-axis sensor, it actually comprises two AMR sensors, one single-axis and one dual-axis magnetometers.

**Figure 1.**
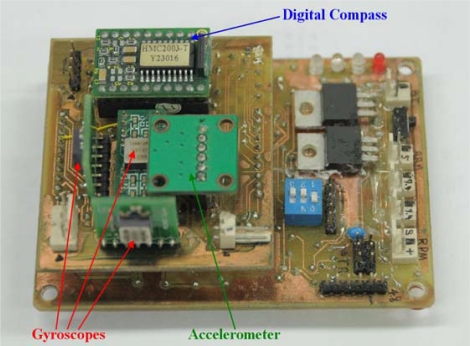
Configuration of the self-developed AHRS.

All these sensors provide analog signals, so an analog-to-digital converter (ADC) is required to acquire the data. Therefore, the PIC18F2553 single-chip microcontroller, made by Microchip Technology, with 10-channel 12-bit ADC is used. In order to increase the computational efficiency and to perform the data fusion algorithm, two PIC18F2553 microcontrollers serve as the processing units of the low-cost AHRS, and they communicate with each other through a built-in Inter-Integrated Circuit (I2C) bus. Moreover, the estimated orientation and the raw data of the AHRS are passed to the personal computer (PC) *via* the universal asynchronous receiver/transmitter (UART) interface. The developed AHRS is low-cost due to the application of low priced sensors and microcontrollers and the implemented data fusion algorithm is self-developed. There is no cost-effective testing of this AHRS, but for this testing readers can be referred to the study in [14]. The configuration of this self-developed AHRS is shown in Figure 1.

### 2.2. Data Fusion Algorithm

In order to achieve the application of the AHRS on the navigation of a small UAV, a data fusion algorithm using the second-order complementary filter to estimate the roll and pitch angles is introduced in this study. This algorithm fuses the data measured from the gyroscope and accelerometer triads to obtain the estimated roll and pitch angles, but there is no information about the yaw angle in these two sensors. Therefore, the digital compass is required to provide the information for the estimation of the yaw angle. Since the gyroscope has the problem of drift which results in cumulative errors, especially for the MEMS sensor, some error compensation for the drift will be necessary to estimate a reliable attitude. This is the reason why the data fusion algorithms use of different type of sensors are required in the attitude estimation of low-cost AHRS [15]. In this study, the Euler angles, namely roll, pitch and yaw angles, are adopted as the orientation representation. The roll and pitch angles are estimated by fusing the outputs of the accelerometer and gyroscopes with a second-order complementary filter [16]. In this filter, the accelerometer serve as an inclinometer to measure the roll and pitch angles under the assumption that the object where the AHRS attached to is not moving or moving in constant speed, hence the gravity is the only source of acceleration acting on the sensors. Under this assumption, the roll and pitch angles can be estimated by the following equations:

(1) $$\phi_{\mathrm{Acc}}={\text{tan}}^{-1}(\frac{a_{y}}{a_{z}})$$

(2) $$\theta_{\mathrm{Acc}}={\text{tan}}^{-1}(\frac{a_{x}}{\sqrt{a_{y}^{2}+a_{z}^{2}}})$$

where *ϕ_Acc_* and *θ_Acc_* are the roll and pitch angles estimated from the accelerometer outputs, respectively; *a_x_*, *a_y_* and *a_z_* are the components of the acceleration measured by accelerometer in the body coordinate frame.

**Figure 2.**
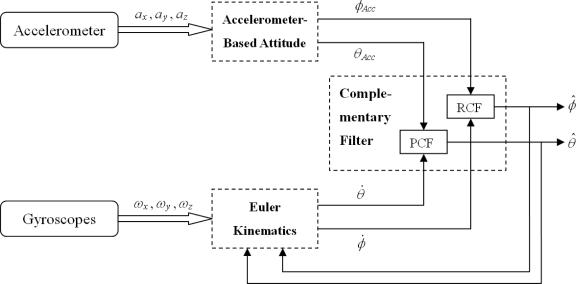
Block diagram of the data fusion algorithm.

The basic idea of the complementary filter is to pass the attitude derived from the gyroscope through a high-pass filter and the attitude derived from the accelerometer through a low-pass filter and then to fuse those signals to obtain the estimated attitude, thus compensating for the drift on the gyroscope and for the slow dynamics of the accelerometer. Consequently, the estimated attitudes would have both short-term and long-term accuracies. Figure 2 shows the block diagram of the data fusion algorithm by using the complementary filter, where *ϕ̂* and *θ̂* are the estimated roll and pitch angles respectively, and *ϕ̇* and *θ̇* are the roll and pitch angle rates respectively, which are transformed from the angular rate measured by the gyroscopes in body coordinate frame into the inertial coordinate frame *via* the Euler Kinematics. The Euler Kinematics is as follows:

(3) $$\left[\begin{array}{c} \dot{\phi }\\ \dot{\theta }\\ \dot{\psi } \end{array}\right]=\left[\begin{array}{ccc} 1 & \text{sin}\;\hat{\phi }\;\text{tan}\;\hat{\theta } & \text{cos}\;\hat{\phi }\;\text{tan}\;\hat{\theta }\\ 0 & \text{cos}\;\hat{\phi } & -\text{sin}\;\hat{\phi }\\ 0 & \text{sin}\;\hat{\phi }\;\text{sec}\;\hat{\theta } & \text{cos}\;\hat{\phi }\;\text{sec}\;\hat{\theta } \end{array}\right]\left[\begin{array}{c} \omega_{x}\\ \omega_{y}\\ \omega_{z} \end{array}\right]$$

where *ω_x_*, *ω_y_* and *ω_z_* are the angular rates measured by the gyroscopes in body coordinate frame. The blocks labeled as “RCF” and “PCF” in Figure 2 represent the roll and pitch complementary filters, respectively. This shows that two parallel complementary filters are required in the data fusion algorithm.

**Figure 3.**
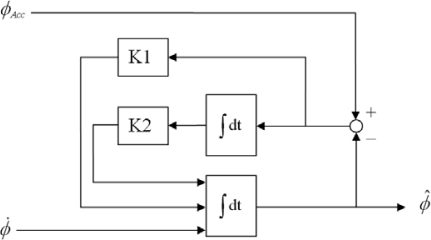
Block diagram of the roll complementary filter.

Figure 3 shows the block diagram of the roll complementary filter, where K1 is the gain for the difference between *ϕ_Acc_* and previously estimated roll angle *ϕ̂,* and K2 is the gain for the integral of this difference. The second-order complementary filter for the roll angle can be depicted by the following transfer function:

(4) $$\hat{\phi }=\frac{\omega_{0}^{2}(2\zeta /\omega_{0})D}{D^{2}+2\zeta \omega_{0}D+\omega_{0}^{2}}\phi_{\mathrm{Acc}}+\frac{D^{2}}{D^{2}+2\zeta \omega_{0}D+\omega_{0}^{2}}\phi$$

where D is the differential operator, *ω*_0_ is the natural frequency, and *ζ* is the damping ratio. The adopted *ω*_0_ and *ζ* in this study are 0.25 and 3.0, respectively. The derivation of this transfer function is described in [16]. The pitch complementary filter is identical to the roll complementary filter mentioned above.

With the estimated roll and pitch angles, the yaw angle can be derived from the measured strength of the magnetic field in body coordinate frame by the digital compass:

(5) $$\psi ={\text{tan}}^{-1}(\frac{m_{z}\;\text{sin}\;\hat{\phi }-m_{x}\;\text{cos}\;\hat{\phi }}{m_{x}\;\text{cos}\;\hat{\theta }+\text{sin}\;\hat{\theta }(m_{y}\;\text{sin}\;\hat{\phi }+m_{z}\;\text{cos}\;\hat{\phi })})$$

where *m_x_*, *m_y_*, and *m_z_* are the components of the magnetic field strength in body coordinate frame.

## 3. Three-Axis Rotating Platform Development

### 3.1. Design of the Platform

**Figure 4.**
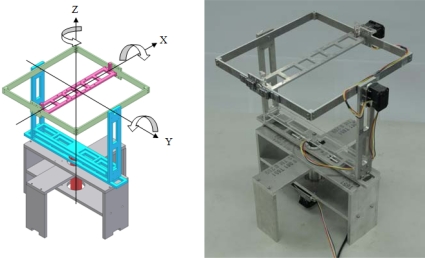
Developed three-axis rotating platform.

In order to calibrate the low-cost sensors and to validate the performance of the AHRS, a platform with three axes of rotation and exact orientation feedback is developed. Since the Earth’s gravity and magnetic fields are both homogenous in specific locations, the calibration of the accelerometer and magnetometer could be executed by the scalar calibration, which is accomplished by rotating the platform to various random orientations. On the other hand, the magnetometer is sensitive to those components with ferromagnetic materials and the wires with high current, the platform should be fabricated with nonmagnetic materials. Moreover, the test section, to which the sensors are attached, should be far away from these sources of interference. Therefore, all the components of the platform are fabricated with aluminum and plastic, and its mechanism is designed as a gimbaled platform as shown in Figure 4. The test section is located in the center of the gimbaled part which is on top of the whole platform and away from the actuators and electrical wires.

The bias calibration of the gyroscope can be executed by fixing the platform in static condition, but the scale factor calibration should be performed under specific rotation for each axis with desired angular rate. In order to achieve this requirement, three axes of rotation are driven individually by high torque servo motors. Two Dynamixel RX-28 motors were installed on the roll and pitch axes, and one Dynamixel RX-64 motor was installed on the yaw axis. These servo motors are produced by the Robotis and capable of providing the angular position feedback with the resolution of 0.29°, which is depicted in the datasheet. From the datasheet, in our case the maximum angular rate for RX-28 is 480°/s and for RX-64 is 320°/s.The range of the angular position feedback of the servo motor is ±150°. In order to achieve the heading angle validation, one incremental encoder with the resolution of 1,024 counts/rev was setup to the z-axis of the platform, hence the range of yaw angular position feedback increases to 360°. These servo motors are controlled by receiving commands from the PC *via* the UART interface to the built-in controller. The received commands include the target angular position and the angular rate for each servo motor. Therefore, every axis of the platform can be rotated with desired angular rates to achieve the scalar calibration of the gyroscope triad.

In order to ensure the manufacturing precision of the platform and to reduce the axial misalignment, all the components are drafted with the computer-aided design (CAD) software and then fabricated by the computer numerical controlled (CNC) machine. The control of the platform and the data acquisition of the sensors are executed by a PC via UART interface with a self-developed user interface, which is composed in Borland C++ Builder (BCB) software. The parameters of the developed three-axis rotating platform are described in Table 1. The estimated hardware cost of this platform is about 1,500 US dollars and for the fabrication it is about 3,000 US dollars.

### 3.2. Calibration of the Platform

After setting up the platform, its calibration was conducted to ensure the accuracy of the angular position feedback. Since the position sensor is the only sensor in the servo motor to provide the angular information, the angular rate is derived from the differentiation of the feedback position. Moreover, the position sensor in yaw axis is the incremental encoder, which is the precision optical sensor. For these reasons, only the calibrations of the roll and pitch axes are demonstrated in this study. The objective of the position calibration is to obtain the relation between the feedback position of the servo motor and the reference angle. The reference angle was acquired from an off-the-shelf AHRS, which is the MTi produced by Xsens. This AHRS provides the angular resolution of 0.05° and the static accuracy below 0.5° for the roll and pitch angles. The calibration procedure is to install the MTi on the test section of the platform and to rotate the roll and pitch axes individually to make two round trips within the range of ±90° as slow as possible. This range is sufficient for the validation of the AHRS and it also can achieve the requirement of the scalar calibration for the sensor triads. The scale factors of the gyroscope triad also could be extracted by making the rate of the platform steady within this range for a period of time.

**Figure 5.**
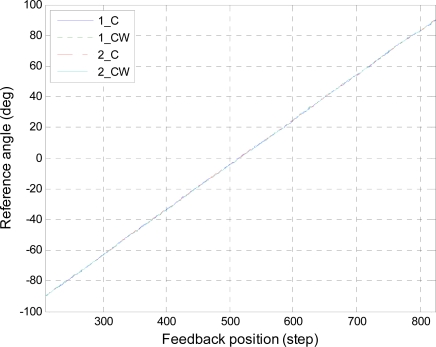
Position calibration for roll axis.

**Figure 6.**
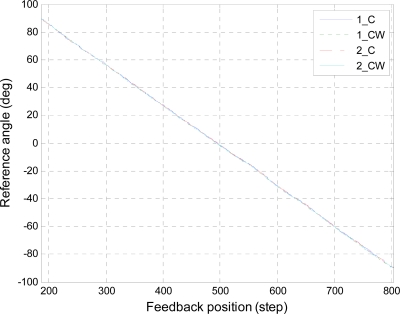
Position calibration for pitch axis.

Figures 5 and 6 show the results of the position calibration for roll and pitch axes, respectively. In these figures, the data denoted as “1_C” and “1_CW” are the clockwise and counterclockwise rotations in first round trip, respectively. The data denoted as “2_C” and “2_CW” are similarly the rotations in second round trip. In order to validate the repeatability of the feedback positions, the polynomial curve fitting of degree one was applying to fit the data. The results of the curve fitting were listed in Table 2 and Table 3 for roll and pitch angles, respectively. The results show that both the slopes and the offsets are close for each axis. Besides, the slopes, which represent the angles per step for the servo motor, correspond with the resolution described in the datasheet. The standard deviations of the errors for each axis are described in Table 4. The errors come from the subtraction between the rotations of different round trips in the same direction. From these results, it is evident that the feedback positions are repeatable and the standard deviations of errors are lower than 0.29° for roll and pitch axes.

### 3.3. Response of the Platform

The performance of the platform can be assessed by examining the step response on each axis individually. The execution of the step response is also the procedure to extract the scale factors of the gyroscopes for the scalar calibration of the gyroscope triad in this study. The scale factor of the gyroscope is the relation between the actual angular rate acting the sensitivity axis and the output value of the gyroscope. Since the rotating rate of the platform can be specified, the angular rate for each axis can be set to a desired value. With the position feedback, the actual angular rates of the platform are derived by using the differentiation of the feedback positions. Therefore, the angular rate of the platform was set to desired value, and then the constant rate for a period of time of the platform was acquired to be the actual rate when executing the step response.

**Figure 7.**
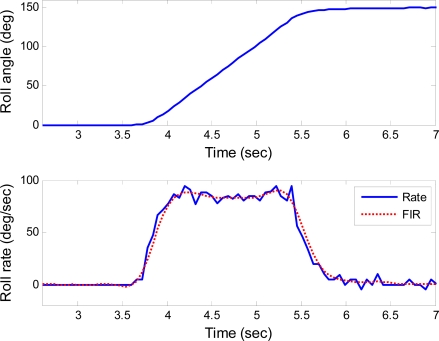
Step response for roll axis.

**Figure 8.**
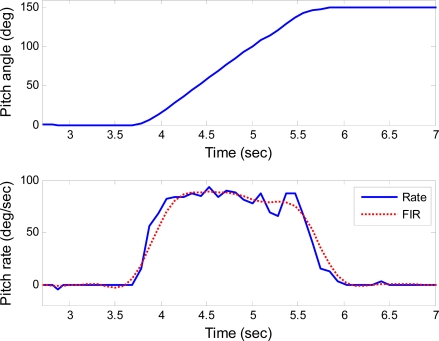
Step response for pitch axis.

**Figure 9.**
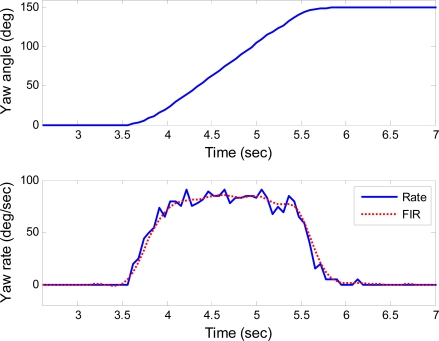
Step response for yaw axis.

The step responses of the roll, pitch and yaw axes are shown in Figures 7, 8 and 9, respectively. These responses were under the rotating rate of 90°/s with the step of 150°. Data denoted as “Rate” and “FIR” are the actual rate derived from the position feedback and its filtering with an low-pass finite impulse response (FIR) filter, respectively. The low-pass FIR filter is required, because the resolution of the position feedback is noisy which will introduce severe error into the differentiation of the feedback positions. The scale factors of the gyroscopes can be measured from these figures, even though the rates do not always keep constant during the step response. For example, when the actual angular rates maintain constant for a period of time as shown in the time range from 4.5 to 5 seconds in Figure 7, the scale factor of the gyroscope in roll axis can be extracted.

## 4. Application of the Rotating Platform to the Sensor Calibrations

### 4.1. Sensor Error Model

The low-cost MEMS and AMR sensors suffer from various errors due to the results of manufacturing imperfections and other effects. These errors can be divided into two categories: random constants and time-correlated random process errors [17]. In this study, the concerned calibration parameters are the random constants, scale factors, biases, and orthogonalization angles. The other errors and effects like the nonlinearities, misalignments, the thermal effects, and the cross-axis effects are neglected in order to simplify the calibration procedures and to realize the error compensation in the low-cost AHRS. The thermal effect is a big issue for the MEMS sensors, and the further study of this effect is shown in [14].

The outputs of the sensors are in voltage, acquired from the ADC in the microprocessors. The default relationship between the output voltage and the physical quantity acting on the sensor sensitivity axis is obtained from the data sheet of different sensor types produced by the manufacturer. In ideal case, the scale factor is equal to the default value on the data sheet and the bias is equal to zero, but this usually is not the case in practice. Actually, the scale factor will deviate from the default value, and the bias is a non-zero value. The scale factor matrix **K** and the bias vector *b⃑* of a sensor triad are modeled as:

(6) $$K=\left[\begin{array}{ccc} k_{x} & 0 & 0\\ 0 & k_{y} & 0\\ 0 & 0 & k_{z} \end{array}\right],\vec{b}=\left[\begin{array}{c} b_{x}\\ b_{y}\\ b_{z} \end{array}\right]$$

**Figure 10.**
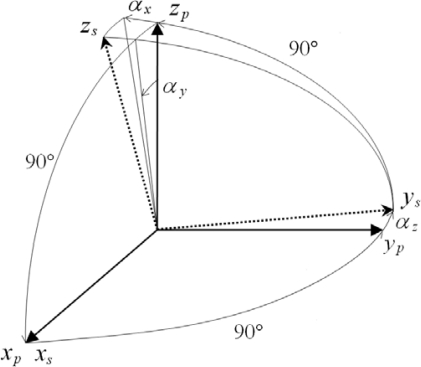
Nonorthogonal angles [4].

The platform coordinate frame is assumed to be orthogonal due to its precise manufacturing and assembly. The nonorthogonal angles are defined by the deviations from the nonorthogonal sensitivity axes of the sensor triad to the orthogonal platform axes with the same origin. It is convenient to define the platform coordinate frame to be the coordinate frame of the orthogonalized sensor triad. In order to reduce the number of the calibration parameters, the x-axis of the platform, *x_p_*, is defined to be identical to the x-axis of the sensitivity axes, *x_s_*, and the *y_p_* is defined to be in the *x_s_y_s_* plane as shown in Figure 10. Moreover, the nonorthogonal angle errors of the corresponding axes are assumed to be small angles. This implies that the sensitivity axes of the sensor triads are nearly orthogonal. With these definitions and assumptions, the nonorthogonal matrix for the specified sensor triad can be derived as follows [4]:

(7) $$T_{s}^{p}=\left[\begin{array}{ccc} 1 & 0 & 0\\ \alpha_{z} & 1 & 0\\ -\alpha_{y} & \alpha_{x} & 1 \end{array}\right]$$

where $T_{s}^{p}$ transforms the nonorthogonal sensitivity axes of the specified sensor triad into the orthogonal platform axes; *a_x_*, *a_y_* and *a_z_*, are the nonorthogonal angles.

The sensor error model of the specified sensor triad is then modeled as follows:

(8) $$\vec{y}={\mathrm{KT}}_{s}^{p}\;\vec{u}+\vec{b}$$

where *y⃑* is the sensor outputs and *u⃑* is the observed physical quantity. This error model is similar to that one, which is a bias and scale factor model, in [18] and it is applied for different sensor triads, which are accelerometer, gyroscope and magnetometer triads in this study.

### 4.2. Calibration Procedures and Results

With the developed platform, the calibration procedures for each sensor triad can be accomplished by rotating the sensors to various random orientations or specified rotations. The estimated physical quantity $\hat{\vec{u}}$ can be derived from Equation (8) as follows:

(9) $$\hat{\vec{u}}={\left(T_{s}^{p}\right)}^{-1}K^{-1}(\vec{y}-\vec{b})$$

For each sensor triad, there are nine parameters, three scale factors, three biases and three nonorthogonal angles, to be determined, so that nine or more orientations and rotations are required to determine these parameters. The adopted method to estimate these parameters is the least squares method. By minimizing the objective function *O*(*p⃑*), the optimization of the parameter vector *p⃑* can be estimated. The objective function is defined as the mean square error between the reference value of the observed physical quantity, *u_ref_*, and the norm of the estimated physical quantity, $u(\vec{p})=\left\|\hat{\vec{u}}\right\|$. That is:

(10) $$O(\vec{p})=\frac{1}{N}\sum_{n=1}^{N}{\left(u_{\mathrm{ref}}-u(\vec{p})\right)}^{2}$$

where *N* is the sum of measured data vectors in desired orientations or rotations. The parameter vector is represented as:

(11) $$\vec{p}=[\begin{array}{ccccccccc} k_{x} & k_{y} & k_{z} & b_{x} & b_{y} & b_{z} & \alpha_{x} & \alpha_{y} & \alpha_{z} \end{array}]$$

Using the method described previously, the calibration of the accelerometer and magnetometer triads can be accomplished by using the scalar calibration method described in [6,7]. This method is based on the fact that the magnitudes of the Earth’s gravity and magnetic fields are constant in a specified location without the influence of the other disturbances; therefore, the reference values of the accelerometer and magnetometer triads are maintained constant and independent of the orientation. For this reason, the exact orientation of the sensor triad is not required. By fixing the sensor triads to at least nine different orientations and applying the least squares optimization, the nine parameter vector can be determined. The calibration results of the accelerometer and magnetometer triads are presented in Table 5 and 6, respectively.

The calibration of the gyroscope triad also can be accomplished by applying the scalar calibration with the platform. The platform can perform the specified rotation about the individual sensitivity axis with constant angular rate while the reference value of the rotating sensor triad can be determined. Moreover, the accuracy biases of the gyroscope triad can be first determined by keeping the platform in static condition. After the biases are determined, the remaining unknown parameters are reduced to six, and it requires at least six different rotations of the platform with constant angular rate to apply the least squares optimization. The result of the gyroscope calibration with this procedure is shown in Table 7.

## 5. Application of the Rotating Platform to the AHRS Calibration and Validation

### 5.1. AHRS Calibration

After completing the sensor calibration, the 27 calibration parameters of three sensor triads are written into the memory of the microprocessor and perform the compensation of the scale factor, bias, and orthogonalization errors for each sensor triad. Also, the data fusion algorithm mentioned in Section 2 is performed in real-time on the microprocessor by using the compensated sensor outputs to estimate the orientation of the AHRS. Due to the time-correlated errors of the sensors and other uncertainties, a second calibration for the outputs of the AHRS is required to compensate the scale factor and bias errors of the estimated Euler angles before the validation. The bias calibration is accomplished by keeping the AHRS level and stationary on the platform, letting its x-axis point to the true north, and then acquiring the biases of the Euler angles. The scale factor calibration is done by rotating the AHRS on the platform and then scaling the AHRS outputs to the correct angles acquired from the platform feedback. The result of the second calibration is shown in Table 8.

### 5.2. AHRS Validation

The purpose of the validation for the developed AHRS is to validate the reliability and practicability of the calibrated sensors and data fusion algorithm. With these calibrated parameters of the sensors and the AHRS, the estimated angle errors can be eliminated or reduced to acceptable region. Two tests, static test and dynamic test, were conducted to demonstrate the AHRS validation in this study. The process of the static test is to maintain the platform steady for a long period of time, which includes about 30 min warming-up duration and 2 h data acquiring duration. The feedback angular positions of the servo motors on the platform are served as the reference values.

**Figure 11.**
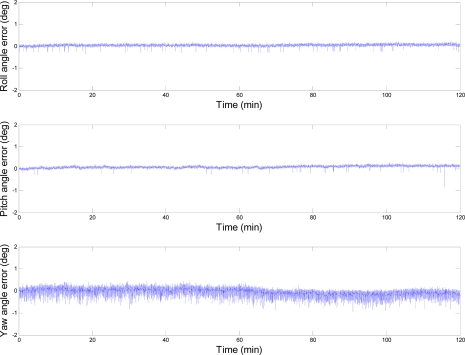
Errors of the AHRS outputs for the static test.

Figure 11 shows the errors of the AHRS outputs, which are the difference between the AHRS outputs and corresponding reference values. The standard deviations of the roll, pitch and yaw angle errors are 0.041°, 0.050° and 0.125°, respectively.

**Figure 12.**
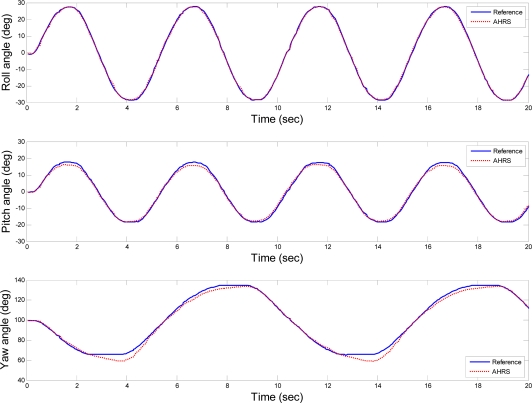
AHRS outputs and the reference values for the dynamic test.

The dynamic test was carried out by rotating the AHRS on the platform and then compared the recorded AHRS outputs with the reference values. The rotations of the platform were achieved by simulating the sinusoid functions for each axis in the user interface and then sending the position commands to the servo motors. The amplitudes of the sinusoid were set to be 30°, 20° and 40° for roll, pitch and yaw axes, respectively. The periods of the sinusoid were set to be 5 s, 5 s and 10 s for roll, pitch and yaw axes, respectively. The errors between the simulated sinusoid functions and reference values are not concerned, since the actual errors of the AHRS are the values between the feedback positions and AHRS outputs. The outputs of the AHRS and the reference values for the dynamic test are represented in Figure 12.

**Figure 13.**
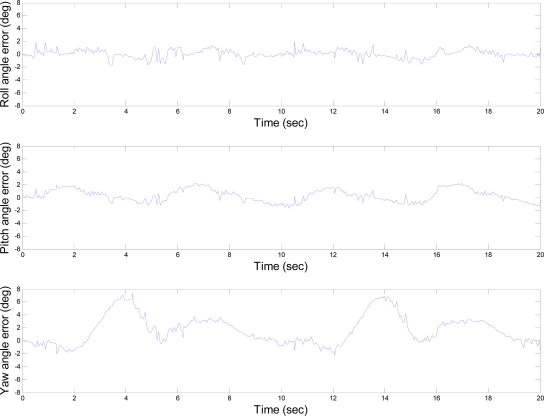
Errors of the AHRS outputs for the dynamic test.

Figure 13 shows the errors of the AHRS outputs, which are the difference between the AHRS outputs and corresponding reference values. The errors in roll, pitch, and yaw angles are within the limits of 2.226°, 2.234° and 7.229°, respectively. The standard deviations of the roll, pitch and yaw angle errors are 0.663°, 0.984° and 2.254°, respectively. Further discussion regarding the results is given in the subsequent section.

## 6. Experimental Results and Discussion

### 6.1. Sensor Calibration Results

From the sensor calibration results in Section 4.2, it is evident that the accelerometer triad contains smaller errors than the other two (see Table 5), since the accelerometer triad consists of one 3-axis sensor while the gyroscope triad consists of three single-axis sensors. Although the digital compass is termed a 3-axis sensor, it actually comprises two AMR sensors, one single-axis and one dual-axis magnetometers as mentioned in Section 2.1. The orthogonalization of the 3-axis sensor is better than the assembled sensor triad with single-axis or dual-axis sensors. Therefore, the magnetometer triad contains larger orthogonalization error than the accelerometer triad. Besides, the Earth’s magnetic field is inevitably contaminated by large distortion, which is caused of the hard iron and soft iron effects [19]. Even though the design of the platform tries to avoid or reduce the influence of the ferromagnetic materials and the wires with high current, the distortion of the Earth’s magnetic field also induced by the nearby ferromagnetic materials in the building and the currents in the electrical wires and circuits of the AHRS. These are the reasons why the orthogonalization error in the magnetometer triad is the biggest.

### 6.2. AHRS Validation Results

From the results of the static test, it is shown that the yaw angle error is larger than the errors in the roll and pitch angles. This is because the yaw angle is derived from the magnetic field which is measured by the digital compass and contains a large noise, as mentioned in previous section. Even so, the errors of the estimated angles from the AHRS are stable and within the acceptable region during the 2-hour data acquisition duration. It demonstrated the stability and reliability of the calibrated parameters and the fusion algorithm.

The results of the dynamic test also show that the yaw angle contains larger error than the other two. This is caused by the more error sources than the other two angles. From Equation (5), it is clear that the yaw angle is derived from the outputs of the digital compass and the roll and pitch angles estimated by the AHRS. The errors in roll and pitch angles will increase the error in yaw, but the major error is caused by the magnetometer outputs, which include the distortion in the Earth’s magnetic field as mentioned in previous section. From Figure 13, one can observe that the errors in pitch and yaw angles are periodical. The periods of these repeated errors are corresponding to those of the applying sinusoids from the platform. A reasonable explanation for this effect is that the origins of each sensor triad do not coincide as shown in Figure 1 and they are not close to the rotating center of the platform. The problem of eccentricity will produce a non-constant angular rate and introduce an unwanted external acceleration to the accelerometer. From above results of the static and dynamic tests, it also shows the practicability of the developed AHRS in real-time.

## 7. Conclusions

A convenient, simple and straightforward procedure for development of a low-cost AHRS has been proposed by using a self-developed three-axis rotating platform. This procedure includes the hardware and software design, sensor calibration, and performance validation. The platform is suitable for the development of low-cost AHRS and it is affordable for most laboratories. The sensor error model, applied to each sensor triad, consists of nine calibration parameters, three scale factors, three biases, and three nonorthogonal angles. The sensor calibration has been accomplished by using the scalar calibration and the least squares methods. The calibrations of the accelerometer and magnetometer triads were executed by fixing the sensors on the platform to various random orientations. The calibration of the gyroscope triad was conducted by rotating the sensors on the platform with specified angular rate. After completing the calibration procedure, the calibrated parameters and the data fusion algorithm for the orientation estimation were implemented to the developed AHRS in real-time. Finally, the validation of the AHRS was demonstrated on the platform. The validation results show that the estimated roll and pitch angles of the developed AHRS are within the acceptable region for most of the practical implementations. The target application of this AHRS is for the navigation of a small UAV. However, the fixed-wing UAV flying at a constant speed suffers induced acceleration when it bank-turns [20]. This results in an unexpected error of the adopted data fusion algorithm. The solution of this problem has also been proposed in [20] by utilizing the low-cost inertial sensors in conjunction with a global positioning system (GPS) sensor. Another solution can be found in [21] by using the fuzzy logic to adapt the parameters of the fused data, which are measured from the accelerometers and gyroscopes.

The proposed procedure validated that the calibration method and data fusion algorithm were successfully implemented for the development of a low-cost AHRS. For implementations that require precise orientation, it is recommended that more errors of the sensor triads and AHRS, such as the nonlinearity, misalignment, and magnetic distortion, *etc.,* be calibrated. The benefit of this study is that all the calibration and validation of the AHRS were accomplished by the same low-cost platform. This means that no other sensor is required after the calibration of the platform itself. Besides, the validation of the AHRS was accomplished on this platform automatically with the control of the user interface, which can simulate the sinusoid functions with different amplitudes and frequencies for each axis. The novelty associated with the study is that the platform may simulate various dynamic motions, which means that the platform can be applied to develop the AHRS for different applications with different dynamics.

## Figures and Tables

**Table 1.** Parameters of the developed platform.

Platform dimensions	398 × 350 × 456 mm^3^
Degrees of freedom	3
Range of motion	360°
Resolution of angular position feedback	0.29°
Maximum rate of motor (roll, pitch)	480°/s
Maximum rate of motor (yaw)	320°/s
Range of angular position feedback (roll, pitch)	±150°
Range of angular position feedback (yaw)	0–360°
Test section area	300 × 300 mm^2^
Maximum load weight	0.6 kg

**Table 2.** Results of the position calibration for roll axis.

Roll axis	Slope (°/step)	Offset (°)
1_C	0.29287	−150.83
1_CW	0.29335	−151.08
2_C	0.29309	−150.95
2_CW	0.29356	−151.23

**Table 3.** Results of the position calibration for pitch axis.

Pitch axis	Slope (°/step)	Offset (°)
1_C	−0.28965	143.33
1_CW	−0.29064	143.42
2_C	−0.28963	143.30
2_CW	−0.29064	143.46

**Table 4.** Standard deviations of errors for each axis.

Axis	Error	Standard deviation (°)
Roll	1_C–2_C	0.2603
	1_CW–2_CW	0.2279
Pitch	1_C–2_C	0.2826
	1_CW–2_CW	0.2204

**Table 5.** Calibrated parameters of the accelerometer triad.

**Axis**	**Scale factor (V/g)**	**Bias (V)**

x	0.349	0
y	0.342	0.025
z	0.326	0.105

**Nonorthogonal angle (°)**	

*α_x_*	0.02
*α_y_*	−0.11
*α_z_*	0.06

**Table 6.** Calibrated parameters of the magnetometer triad.

**Axis**	**Scale factor (V/gauss)**	**Bias (V)**

x	1.008	0.205
y	1.040	−0.108
z	1.036	0.032

**Nonorthogonal angle (°)**	

*α_x_*	−6.69
*α_y_*	−3.90
*α_z_*	1.33

**Table 7.** Calibrated parameters of the gyroscope triad.

**Axis**	**Scale factor (mV/°/s)**	**Bias (V)**

x	5.134	−0.038
y	5.547	0.053
z	5.515	−0.037

**Nonorthogonal angle (°)**	

*α_x_*	0.63
*α_y_*	−2.87
*α_z_*	2.34

**Table 8.** Calibrated parameters of the AHRS output angles.

**Euler angle**	**Scale factor ratio**	**Bias (°)**

roll	1.050	−3.5
pitch	1.080	−0.7
yaw	−0.935	0
